# P-2038. A Pharmacist-Run Treatment Decision Program for High-Risk Outpatients with Mild-Moderate COVID-19 at an Urban Veteran's Affairs Medical Center

**DOI:** 10.1093/ofid/ofae631.2194

**Published:** 2025-01-29

**Authors:** Ryan Kuhn, Lauren Weslosky, Suganthini Krishnan Natesan, Sorabh Dhar

**Affiliations:** John D Dingell VA Medical Center, Detroit, Michigan; John D. Dingell VA Medical Center, Troy, Michigan; John D. Dingell VA Medical Center, Wayne State University, OAKLAND TOWNSHIP, Michigan; Wayne State University/Detroit Medical Center, John Dingell VAMC, Detroit, Michigan

## Abstract

**Background:**

Antiviral treatment is recommended for high-risk outpatients with mild-moderate COVID-19 to prevent progression to severe disease. Antiviral treatment decisions remain challenging due to the need for early initiation of treatment and the potential for drug-drug interactions with first-line therapy.

**Methods:**

This single center, retrospective review evaluated the impact of a Pharmacist-run treatment decision program for high-risk outpatients with mild-moderate COVID-19 from 1/1/2022 through 12/28/2023. All outpatient treatment candidates were reviewed by a Clinical Pharmacist prior to initiation of oral antiviral treatment. A 5-day course of oral ritonavir-boosted nirmatrelvir (RNM) was considered first-line. A 5-day course of oral molnupiravir (MNP) was used as an alternative in situations where oral RNM was not clinically appropriate. The Clinical Pharmacist determined the optimal antiviral treatment, the appropriate medication dose, reviewed for drug-drug interactions, helped implement any necessary medication adjustments and/or monitoring plans, and assisted with patient education. Patient characteristics and the number of pharmacist interventions related to RNM drug-drug interactions were summarized.

**Results:**

From 1/1/2022 to 12/28/2023, 455 high-risk outpatients were treated with oral antivirals for mild-moderate COVID-19 through the Pharmacist-run treatment decision program. Four-hundred and two patients (88.35%) were treated with RNM and 53 patients (11.65%) were treated with MNP. Patient ages ranged from 23 to 97 years (mean 66.4, SD 12.1). The mean duration of COVID-19 symptoms for patients who received oral antiviral treatment was 3 days (SD 1.12). The Clinical Pharmacist intervened on a total of 624 drug-drug interactions related RNM. The most common reason for use of MNP was the presence of major drug-drug interactions related to RNM (47/53; 88.7%), followed by severe renal impairment (6/53; 11.3%).

**Conclusion:**

A Pharmacist-run treatment decision program for high-risk outpatients with mild-moderate COVID-19 was effective at initiating appropriate oral antiviral treatment in a timely fashion and ensuring patient safety by managing drug-drug interactions related to RNM.

**Disclosures:**

All Authors: No reported disclosures

